# Change in newly diagnosed Graves’ disease phenotype between the twentieth and the twenty-first centuries: meta-analysis and meta-regression

**DOI:** 10.1007/s40618-020-01479-z

**Published:** 2020-12-21

**Authors:** S. Ippolito, C. Cusini, P. Lasalvia, F. Gianfagna, G. Veronesi, D. Gallo, E. Masiello, P. Premoli, J. Sabatino, A. Mercuriali, A. Lai, E. Piantanida, M. L. Tanda, L. Bartalena

**Affiliations:** 1 Department of Medicine and Surgery, University of Insubria, 21100 Varese, Italy; 2 Research Centre in Epidemiology and Preventive Medicine (EPIMED), University of Insubria, 21100 Varese, Italy; 3 Mediterranea Cardiocentro, 80122 Napoli, Italy; 4 Department of Medicine and Surgery, University of Insubria, Endocrine Unit, ASST dei Sette Laghi, Viale L. Borri, 57, 21100 Varese, Italy

**Keywords:** Graves’ disease, Hyperthyroidism, Goiter, Graves’ orbitopathy, Autoimmunity

## Abstract

**Purpose:**

According to a few recent studies, the clinical phenotype of Graves’ disease (GD) at onset is becoming milder in recent years, in terms of prevalence and severity of hyperthyroidism, goiter and overt eye disease. The aim of this study was to assess the change in GD phenotype across the late twentieth and the early twenty-first centuries.

**Materials and methods:**

We carried out a systematic search of studies published between 1/1/1980 and 12/31/2017 describing naïve GD patients at diagnosis. We collected epidemiological, clinical, biochemical and serological data reported in the selected studies, and (1) conducted a single-arm meta-analysis to compare clinical and biochemical characteristics of naïve GD patients before and after year 2000 and (2) performed a meta-regression to identify the trend of the observed clinical presentations.

**Results:**

Eighty selected articles were related to the period before the year 2000, 30 to the years 2000–2017. According to demographics, the two defined populations were homogeneous at meta-analysis: overall estimated female prevalence was 81% [95% CI 79–82], mean estimated age of the entire population was 39.8 years [95% CI 38.4–41.1], with no significant differences between pre- and post-2000 groups (*p* > 0.05). The overall estimated prevalence of smokers was 40% [95% CI 33–46], with no significant difference between the two groups (*p* > 0.05). Mean estimated free thyroxine (FT4) and free triiodothyronine (FT3) levels at diagnosis were higher in the pre-2000 group: 4.7 ng/dl [95% CI 4.5–4.9] for FT4 and 14.2 pg/ml [95% CI 13.3–15.1] for FT3, as compared to the post-2000 group: 3.9 ng/dl [95% CI 3.6–4.2] for FT4 and 12.1 pg/ml [95% CI 11.0–13.3] for FT3 (all *p* < 0.01). Goiter estimated prevalence was higher in the pre-2000 group, 87% [95% CI 84–90], than in the post-2000 group, 56% [95% CI 45–67]. Estimated prevalence for Graves’ Orbitopathy (GO) was 34% [95% CI 27–41] in the pre-2000 group and 25% [95% CI 19–30] in the post-2000 group (*p* = 0.03). Accordingly, meta-regression adjusted for covariates showed an average annual reduction of FT4 (− 0.040 ± 0.008 ng/dl, *p* < 0.0001), FT3 (− 0.316 ± 0.019 pg/ml, *p* < 0.0001), goiter prevalence (− 0.023 ± 0.008%, *p* = 0.006), and goiter size (− 0.560 ± 0.031 ml, *p* < 0.0001).

**Conclusions:**

Our meta-analysis and meta-regression confirmed that GD phenotype at diagnosis is nowadays milder than in the past; we hypothesize that conceivable factors involved in this change are iodoprophylaxis, worldwide decrease in smoking habits, larger use of contraceptive pill and micronutrient supplementation, as well as earlier diagnosis and management.

**Supplementary Information:**

The online version contains supplementary material available at 10.1007/s40618-020-01479-z.

## Introduction

Graves’ disease (GD) is the most common cause of hyperthyroidism in iodine-sufficient areas [1], ultimately caused by antibodies directed against the TSH-receptor (TRAb). Cumulative lifetime risk to develop GD is 3% for women and 0.5% for men [2], with an incidence of 20–50 cases/100,000 people per year [3]. GD occurs at any age, with the highest risk of onset in the 3rd–5th decades of life, and a female-to-male ratio between 4:1 and 10:1 [4, 5]. TRAb are responsible for the main clinical manifestations of the disease: hyperthyroidism, goiter and orbitopathy, the so-called “Merseburg triad”, by the name of the German city in which the physician Karl von Basedow practiced and described the clinical pattern of this disease [6]. Graves’ orbitopathy (GO) incidence is reported to be 16 cases per 100,000 per year in females and 3 cases per 100,000 in males [7]. Besides GO, rare extrathyroidal manifestations include thyroid dermopathy (or pretibial myxedema) and acropachy [8].

GD is easily diagnosed if hyperthyroidism is associated to extrathyroidal manifestations; diagnosis is more challenging if hyperthyroidism is mild, goiter is absent or multinodular, extrathyroidal manifestations are lacking. In those cases, diagnosis is mainly based on TRAb detection, eventually corroborated by color-flow Doppler ultrasonography, thyroid scintigraphy and/or thyroid uptake of radioactive iodine [9].

Clinical GD features at diagnosis can be heterogeneous, and the clinical phenotype at onset may have changed in the last decades as compared to the classical pattern described in the Merseburg paradigm. According to our recent clinical experience, GD phenotype at diagnosis seems milder in recent years as compared to previous decades. This impression is corroborated by a few articles published in the latest decades, suggesting a lower incidence and severity of GD features as compared to frequencies reported in the literature. In particular, in a 2016 article from two Northern Italy centers (ours, based in Varese, and another based in Pavia), a significant proportion of GD patients at diagnosis presented with mild or moderate GD, about half of them presenting normal thyroid volume, and only 20% presenting GO [10].

To further investigate these preliminary data, aiming to define if the change in clinical presentation between centuries was significant, we first conducted a single-arm meta-analysis to compare clinical and biochemical characteristics in naïve GD patients before and after the year 2000; then, we employed meta-regression to identify the determinants of the observed clinical presentations, among the sample characteristics reported in the primary studies, taking into account the study-specific designs.

## Materials and methods

### Search strategy

In this systematic review and meta-analysis, we adopted procedures consistent with the PRISMA (Preferred Reporting Items for systematic reviews and meta-analyses) [11] and the MOOSE (Meta-analysis of Observational Studies) [12] guidelines. Two authors (C.C. and S.I.) independently searched the online databases MEDLINE (PubMed), Embase, Google Scholar, and the Cochrane Central Register of Controlled Trials, using “Graves’ disease” and synonyms, restricting to the keyword fields whenever available in the bibliographic database. We applied the most inclusive search strategy, considering any type of study (observational studies, and randomized controlled trials, including also those reporting retrospective analyses) and studies in any language, published between 1/1/1980 and 12/31/2017. Studies published after 1980 reporting data from an earlier recruitment period were also included, consequently, data reported in this article start from 1972. The search strategy was refined with manual searches of reference lists.

### Study selection

Studies were eligible for inclusion if they described the clinical, biochemical or serological features of patients with newly diagnosed GD. We excluded: (1) studies reporting the features of patients who had already been treated for GD, (2) studies with inclusion and/or exclusion criteria that select a subset of patients with newly diagnosed GD (e.g., only patients with large goiter), (3) case reports, and (4) studies with overlapping data.

Two authors (C.C. and S.I.) independently selected potentially eligible studies for inclusion. Non-relevant articles were excluded based on title and abstract and duplicates were removed. The full text of the remaining eligible papers was examined in detail to determine their inclusion. Disagreements were solved by discussion between the two authors.

The list of articles included for analysis is detailed in Supplemental Table 1.

### Data collection

Two authors (C.C. and S.I.) extracted the following data from the included studies in a piloted data extraction form: (1) author, publication year, start year, end year, study design; (2) number of patients, gender, ethnicity, mean age, smoking habits; (3) biochemical severity of hyperthyroidism: free thyroxine (FT4) and free triiodothyronine (FT3) levels; (4) autoantibody profile: positivity for TRAb and their levels, positivity for thyroid peroxidase antibody (TPOAb); (5) presence of GO and GO severity; and (6) presence of goiter (clinical and/or ultrasonographic), goiter volume.

We considered each subgroup as a different population if a study reported separately data from different subgroups of patients (e.g., stratification according to patients’ age). In data analyses, we considered the central year of the recruitment period to allocate the studies before or after 2000, or the publication year when recruitment period was not reported.

Prevalence for dichotomous data or mean and standard error for continuous data, along with their sample size, were used for analyses. When data were available only as median and interquartile range, mean and standard deviation were estimated [13] to allow the inclusion in the meta-analysis. Thyroid function tests (FT4, FT3) were evaluated as raw data, suitably converted into ng/dl (FT4) or pg/ml (FT3), and then adjusted for upper limit of normal (ULN) of the given reference range (laboratory value/ULN), to reduce potential bias related to the measurement method. Similarly, TRAb was assessed in terms of prevalence (% positive) and adjusted for upper limit of normal of the given kit. TPOAb was assessed in terms of prevalence (% positive). Goiter at diagnosis was investigated in terms of prevalence and volume, assessed either by palpation (according or analogue to WHO classification [14]) or by ultrasound thyroid lobe diameter measurement, calculated using the formula for a prolate ellipse: (volume = 0.5 {length x depth x width}), and considering 18 ml for males and 14 ml for females as cutoffs to identify goiter presence. Orbitopathy was diagnosed according to different standards in the published articles, either according to clinical evaluation (considering exophthalmos, diplopia or impaired vision), or using standardized criteria, i.e., EUGOGO [15] or NOSPECS [16] classification; due to the high heterogeneity we decided not to compare GO severity between studies, but to merely analyze the overall GO prevalence for each study.

### Outcomes

In the meta-analysis, the primary endpoint was to evaluate the change in the phenotype of newly diagnosed GD between the last decades of the twentieth century (1972–1999) and the first years of the twenty-first century (2000–2017). The year 2000 as cutoff was chosen arbitrarily, aiming to divide in half a time span from the 80s (year from which most studies were available) up-to-date. Factors considered to evaluate the phenotype were: severity of hyperthyroidism, prevalence of GO and its severity, prevalence of goiter and its volume, autoantibody prevalence and titer. Using a meta-regression, the secondary endpoint was to evaluate if there was a progressive trend in the change of these features from 1972 to 2017, using patient- and study-specific characteristics as potential confounding variables.

### Statistical analysis

#### Meta-analysis

Studies computation was performed using the Cochrane Collaboration Review Manager Software (RevMan version 5.3). For meta-analyses, the random-effects methods was used, according to DerSimonian and Laird [17], to take into account potential heterogeneity between studies. For each meta-analysis, between study heterogeneity was assessed using *I*^2^, with values higher than 0 indicating presence of heterogeneity. The analyses were stratified for recruiting years, pre- and post-2000. Test for subgroups differences were tested using Chi-square.

#### Meta-regression

A meta-regression was performed using a linear mixed regression using SAS software (SAS Institute, Cary—NC, version 9.4), considering fixed and random-effect models. Univariate and multivariate analysis were performed, adjusting for age, sex, ethnicity, year of study (median year if recruiting was conducted in more than one year or publication year if recruiting period was not available), and therapeutical indication (none vs. selected therapeutical indication in the original paper).

## Results

### Included studies

The literature search using the strategy mentioned above yielded 12,765 papers. All these records were screened in two steps and assessed for eligibility. One hundred and ten papers provided sufficient quantitative data and were included in the meta-analysis. Eighty papers were included in the pre-2000 group (73 papers published between 1/1/1980 and 12/31/1999, and 7 papers published after 1/1/2000 but referring to studies conducted—completely or mainly—before 2000), 30 papers conducted and published between 1/1/2000 and 12/31/2017 were included in the post-2000 group.

**Fig. 1:**
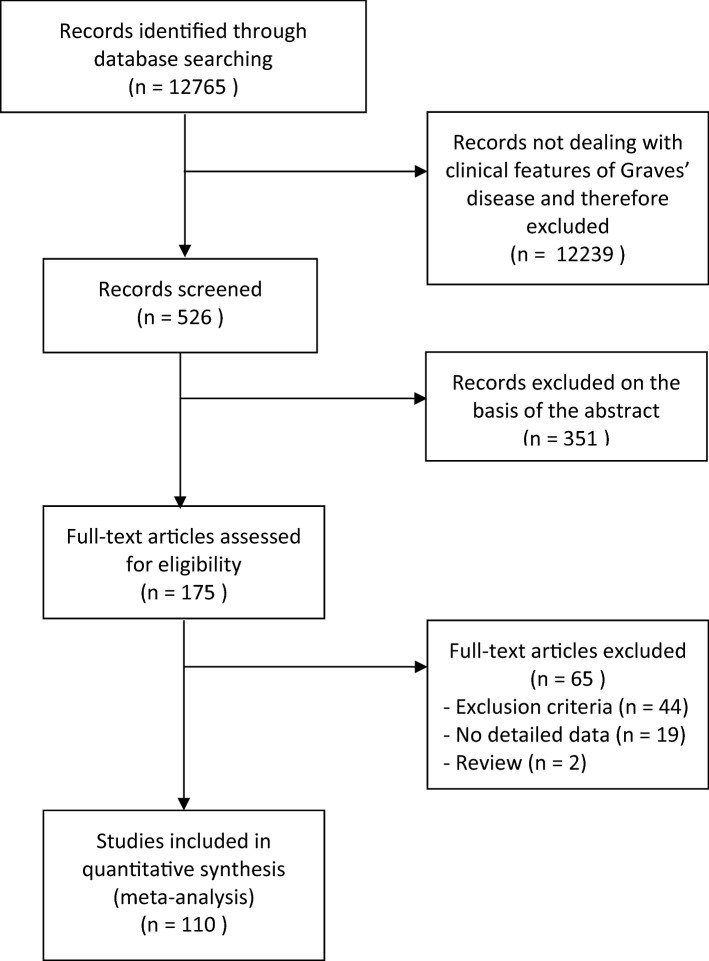
Search strategy and flow diagram according to PRISMA (Preferred Reporting Items for systematic reviews and meta-analyses) (11)

Flow-chart of study selection strategy is depicted in Fig. 1.

**Fig. 2:**
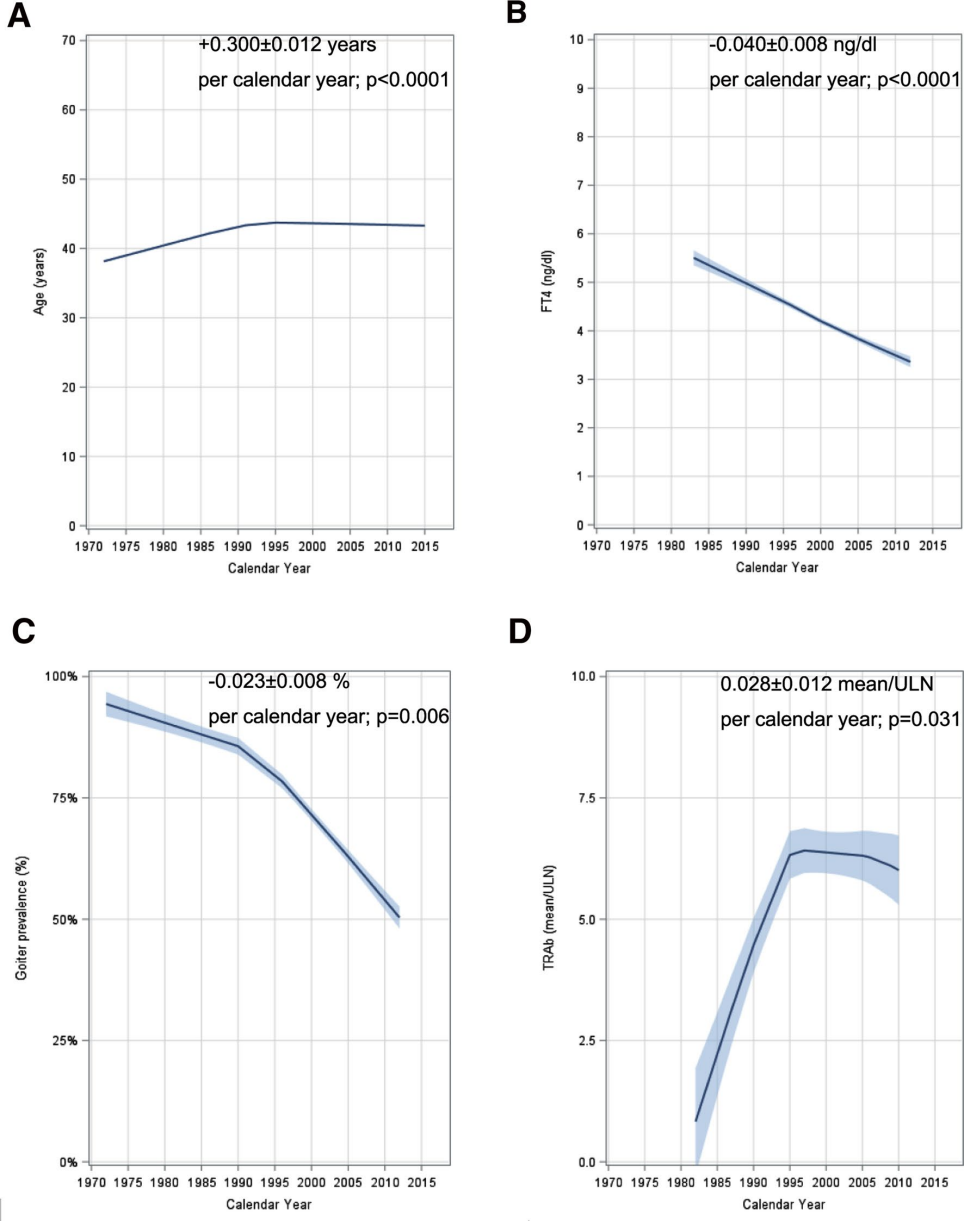
Trends of Graves’ disease (GD) patients’ features at diagnosis throughout the years, from 1972 to 2017. **a** GD patients’ mean estimated age per calendar year. **b** GD patients’ mean estimated free thyroxine (FT4) levels per calendar year. **c** GD patients’ goiter prevalence per calendar year. **d** GD patients’ mean estimated TRAb levels (mean estimated TRAb/upper limit of normal) per calendar year. Smoothened regression using the SAS procedure SGPLOT and LOESS statement with smoothening option fixed to 1, on pooled random databases with mean and SE parameter values of primary studies

A summary of all the meta-analysis performed is provided in Table 1, whereas meta-analyses details and forest plots are available in a dedicated data-repository [18]; meta-regression results are depicted in Table 2 and Fig. 2.

**Table 1:** Meta-analysis of Graves’ disease (GD) phenotype change

Subgroup	Weight	Estimate mean *or* estimate proportion (95% CI)	*I*^2^	Graphic summary^a^
*FT4 (ng/dl)*				
Pre-2000	60.7%	4.7 (4.5–4.9)	97%	
Post-2000	39.3%	3.9 (3.6–4.2)	96%	
Total	100%	4.4 (4.2–4.5)	97%	
Test for subgroup differences: Chi^2^ = 14.84, *p* = 0.0001, *I*^2^ = 93.3%				
*FT3 (pg/ml)*				
Pre-2000	58.4%	14.2 (13.3–15.1)	94%	
Post-2000	41.6%	12.1 (11.0–13.3)	97%	
Total	100%	13.5 (12.6–14.4)	98%	
Test for subgroup differences: Chi^2^ = 7.60, *p* = 0.006, *I*^2^ = 86.8%				
*Goiter prevalence (%)*				
Pre-2000	47.9%	87 (84–90)	75%	
Post-2000	52.1%	56 (45–67)	98%	
Total	100%	70 (64–76)	98%	
Test for subgroup differences: Chi^2^ = 30.37, *p* < 0.00001, *I*^2^ = 96.7%				
*Goiter volume (ml)*				
Pre-2000	62.6%	42.9 (37.2–48.5)	98%	
Post-2000	37.4%	36.8 (32.6–41.1)	98%	
Total	100%	40.7 (36.6–44.7)	99%	
Test for subgroup differences: Chi^2^ = 2.78, *p* = 0.1, *I*^2^ = 64.0%				
*Orbitopathy prevalence (%)*				
Pre-2000	58.7%	34 (27–41)	92%	
Post-2000	41.3%	25 (19–30)	96%	
Total	100%	30 (26–35)	95%	
Test for subgroup differences: Chi^2^ = 4.59, *p* = 0.03, *I*^2^ = 78.2%				
*TRAb prevalence (%)*				
Pre-2000	77.9%	72 (67–78)	88%	
Post-2000	22.1%	88 (83–93)	95%	
Total	100%	76 (72–80)	93%	
Test for subgroup differences: Chi^2^ = 17.42, *p* < 0.0001, *I*^2^ = 94.3%				

Comparison of patients with newly diagnosed GD pre- and post-2000

*FT4* free thyroxine, *FT3* free triiodothyronine, *TRAb* TSH-receptor antibodies

^a^Graphic summary resume meta-analysis results, which are depicted in detail in Supplemental Figures [18]

**Table 2:** Meta-regression results (fixed-effect model)

	Age		FT4 (ng/dl)		FT4 (mean/ULN)		FT3 (pg/ml)		Goiter prevalence (%)	
	*β* ± SE	*p* value	*β* ± SE	*p* value	*β* ± SE	*p* value	*β* ± SE	*p* value	*β* ± SE	*p* value
*Univariate analysis*										
Calendar year	0.279 ± 0.010	** < 0.0001**	− 0.044 ± 0.007	** < 0.0001**	− 0.054 ± 0.006	** < 0.0001**	− 0.318 ± 0.017	** < 0.0001**	− 0.018 ± 0.006	**0.006**
*Multivariate analysis*										
Calendar year	0.300 ± 0.012	** < 0.0001**	− 0.040 ± 0.008	** < 0.0001**	− 0.039 ± 0.007	** < 0.0001**	− 0.316 ± 0.019	** < 0.0001**	− 0.023 ± 0.008	**0.006**
Therapeutical indication	0.663 ± 0.236	**0.006**	0.305 ± 0.102	**0.004**	0.362 ± 0.105	**0.001**	− 0.633 ± 0.272	**0.026**	− 0.059 ± 0.092	0.523
Sex	0.018 ± 0.008	**0.024**	0.006 ± 0.003	**0.037**	− 0.005 ± 0.004	**0.204**	0.050 ± 0.006	** < 0.0001**	0.003 ± 0.003	0.343
Age			− 0.030 ± 0.009	**0.002**	− 0.008 ± 0.012	**0.516**	− 0.081 ± 0.025	**0.002**	0.016 ± 0.008	0.065
Ethnicity	8.554 ± 0.203	** < 0.0001**	− 0.080 ± 0.111	**0.474**	− 0.375 ± 0.119	**0.002**	− 2.824 ± 0.299	** < 0.0001**	− 0.190 ± 0.109	0.094
Records	142		66		65		42		29	

	Goiter volume (ml)		GO prevalence (%)		TRAb (%)		TRAb (mean/ULN)		TPOAb (%)	
	*β* ± SE	*p* value	*β* ± SE	*p* value	*β* ± SE	*p* value	*β* ± SE	*p* value	*β* ± SE	*p* value
*Univariate analysis*										
Calendar year	− 0.729 ± 0.027	** < 0.0001**	− 0.001 ± 0.004	0.746	0.009 ± 0.004	**0.016**	0.044 ± 0.011	**0.0002**	− 0.006 ± 0.005	0.207
*Multivariate analysis*										
Calendar year	− 0.560 ± 0.031	** < 0.0001**	0.001 ± 0.005	0.857	0.009 ± 0.005	0.109	0.028 ± 0.012	**0.031**	− 0.009 ± 0.007	0.195
Therapeutical indication	7.226 ± 0.576	** < 0.0001**	− 0.001 ± 0.090	**0.996**	0.028 ± 0.110	**0.801**	− 0.937 ± 0.282	**0.002**	− 0.048 ± 0.124	0.705
Sex	0.136 ± 0.016	** < 0.0001**	0.0004 ± 0.002	**0.821**	− 0.0003 ± 0.003	**0.916**	0.002 ± 0.010	**0.825**	0.002 ± 0.003	0.365
Age	0.300 ± 0.065	** < 0.0001**	− 0.004 ± 0.005	**0.475**	0.006 ± 0.008	**0.489**	− 0.224 ± 0.025	** < 0.0001**	0.005 ± 0.006	0.419
Ethnicity	− 8.514 ± 0.803	** < 0.0001**	− 0.089 ± 0.078	**0.263**	0.015 ± 0.077	**0.852**	− 0.968 ± 0.365	**0.011**	− 0.033 ± 0.084	0.697
Records	32		45		35		48		35	

Change of Graves’ disease phenotype at diagnosis by year, from 1972 to 2017, univariate and multivariate analyses. Fixed- and random-effect model results were similar; however, fixed-effect model results were reported due to reliability of explanatory betas

### Demographics

Considering separately each subgroup of patients described in the studies selected, the pre-2000 group included 162 entries (15,535 patients) and the post-2000 group 49 entries (6868 patients).

According to demographics, the two defined populations resulted homogeneous at meta-analysis. Gender and ethnicity estimated proportion were not different between groups: 81% [95% CI 79–83] of females and 80% [95% CI 79–81] of Caucasians in both pre- and post-2000 groups (*p* = 0.93 and *p* = 0.51 at test for subgroup differences, for gender and ethnicity proportion, respectively, Supplemental Fig. 1 and 2 [18]); mean age of the entire population was 39.8 years [95% CI 38.4–41.1] with no difference (*p* = 0.25) between the pre-2000 group (*n* = 14,086, mean age 39.4 years [95% CI 37.7–41.1]) and the post-2000 group (*n* = 5946, mean age 40.9 years [95% CI 39.0–42.7], Supplemental Fig. 3 [18]), yet the meta-regression showed a minor, but significant, increase of age at diagnosis over time (0.300 ± 0.012 per year, *p* < 0.0001, Table 2 and Fig. 2a). The overall estimated prevalence of smokers was 40% [95% CI 33–46], with no significant differences between the two groups (*p* = 0.62; pre-2000 group: *n* = 1172, 41% [95% CI 33–50]; post-2000 group: *n* = 3902, 38% [95% CI 28–48], Supplemental Fig. 4 [18]) at meta-analysis.

### Hyperthyroidism

FT4 estimated mean values at diagnosis were significantly lower (*p* = 0.0001, Table 1 and Supplemental Fig. 5 [18]) in the post-2000 group (*n* = 3146, mean FT4 3.9 ng/dl [95% CI 3.6–4.2]) than the pre-2000 group (*n* = 2711, mean FT4 4.7 ng/dl [95% CI 4.5–4.9]). This result was confirmed using FT4 levels adjusted for upper limit of normal (ULN) of the given reference range (FT4/ULN), to reduce potential bias related to the measurement method: estimated mean ratio was lower in the post-2000 group (2.2 [95% CI 2.1–2.4]) as compared to the pre-2000 group (2.6 [95% CI 2.3–2.9]), *p* = 0.03 at test for subgroups differences (Supplemental Fig. 6 [18]). Indeed, meta-regression demonstrated a progressive FT4 reduction over the years (− 0.040 ± 0.008 ng/dl per year, *p* < 0.0001, Table 2 and Fig. 2b).

Likewise, FT3 estimated mean was significantly lower in the post-2000 group (*n* = 3476, mean  FT3 12.1 pg/ml [95% CI 11.0–13.3]) as compared to the pre-2000 group (*n* = 4069, mean FT3 14.2 pg/ml [95% CI 13.3–15.1]), *p* = 0.006 at test for subgroup differences (Table 1 and Supplemental Fig. 7 [18]).

### Goiter

Goiter overall estimated prevalence was 70% [95% CI 64–76]; this prevalence was significantly lower (*p* < 0.00001, Table 1 and Supplemental Fig. 8 [18]) in the post-2000 group (*n* = 2566, mean prevalence 56% [95% CI 45–67]) compared to the pre-2000 group (*n* = 3101, mean prevalence 87% [95% CI 84–90]). In terms of volume, we observed a trend toward a reduced volume in the post-2000 group (*n* = 1775, mean volume 36.8 ml [95% CI 32.6–41.1]) compared to the pre-2000 group (*n* = 1345, mean volume 42.9 ml [95% CI 37.2–48.5]), though not reaching statistical significance (*p* = 0.1, Table 1 and Supplemental Fig. 9 [18]). Indeed, meta-regression demonstrated a progressive goiter reduction both in terms of prevalence (− 0.023 ± 0.008% per year, *p* = 0.006) and in terms of volume (− 0.560 ± 0.031 ml per year, *p* < 0.0001, Table 2 and Fig. 2c).

### Graves’ orbitopathy

GO overall estimate prevalence was 30% [95% CI 26–35], with a significant lower estimate in the post-2000 group (*n* = 4486, mean prevalence 25% [95% CI 19–30]) than in the pre-2000 group (*n* = 2258, mean prevalence 34% [95% CI 27–41]), *p* = 0.03 at test for subgroups differences (Table 1 and Supplemental Fig. 10 [18]). At meta-regression analysis, GO prevalence did not show a significant change per year (0.001 ± 0.005 per year, *p* = 0.857, Table 2).

### Autoimmunity

TRAb laboratory measurement was clearly heterogeneous among different studies and different years. Therefore, we partially overcame this bias evaluating TRAb positivity prevalence and adjusting antibodies level for the upper limit of normal. We found a significant increase in the estimated prevalence in the post-2000 group (*n* = 2175, mean prevalence 88% [95% CI 83–93]) than in the pre-2000 group (*n* = 2095, mean prevalence 72% [95% CI 67–78]), *p* < 0.0001 at test for subgroup differences (Table 1 and Supplemental Fig. 11 [18]); indeed, a mild increase through years in both antibodies prevalence and adjusted serum levels was found at meta-regression study, though at multivariate analysis only serum-adjusted levels reached statistical significance (0.009 ± 0.005 per year, *p* = 0.109 for TRAb prevalence; 0.028 ± 0.012 per year, *p* = 0.031 for TRAb serum levels adjusted for upper limit of normal, Table 2, Fig. 2d).

TPO prevalence was similar between in the pre- and post-2000 groups (pre-2000: *n* = 2785, mean prevalence 75% [95% CI 70–80]; post-2000: *n* = 1156, mean prevalence 68% [95% CI 58–79]), *p* = 0.27 for subgroup differences (Supplemental Fig. 12 [18]).

## Discussion

GD is an autoimmune disease, ultimately caused by TSH-receptor antibodies targeting thyrocytes, and thereby causing, in most cases, thyroid hyperplasia, hyperthyroidism and extrathyroidal manifestations. GD pathogenesis is complex and not fully understood yet, and involves both genetic and environmental factors [2], accounting for the differences in clinical presentation among patients and, possibly, for a phenotype change through decades. Although the Merseburg triad is still characterizing many cases of GD at presentation, severity and prevalence of these manifestations seem less prominent in latest years. To further explore this issue, our analysis was conducted analyzing articles published from 1980 to 2017, describing naïve, untreated GD patients’ features at diagnosis.

Demographic characteristics were similar between the two centuries and comparable to the literature [19]: female sex was predominant as expected, with a pooled prevalence of 80%. Interestingly, mean age of the overall cohort was about 40 years, with no strong differences between groups at meta-analysis but showing a minor, though significant, increase in age at GD diagnosis per year at meta-regression: this outcome supports the hypothesis that the change in GD phenotype is not exclusively due to an earlier diagnosis: in fact, if that were the case, patients at diagnosis should have been younger, whereas they have similar, if not slightly older, age.

Smoking prevalence, the main environmental risk factor for GD and especially GO [20], did not change significantly between the pre-2000 and post-2000 cohorts, the pooled estimated prevalence of smokers in the overall GD cohort was 40%. At a first glance, this proportion seems superimposable to the global prevalence of smoking, since nearly 47% of men and 12% of women smoke [21]; however, we must take into account that smoking prevalence is much higher in males, whereas GD is greatly predominant in females, confirming previous findings showing that smokers are overrepresented amongst patients with GD [20]. Therefore, it is challenging to determine if and how smoking has an impact in GD phenotype modifications; an intriguing hypothesis is that a decrease, if not in prevalence at least in the number of cigarettes smoked per day, and the smoking restrictions in public locations made by most developed countries, with consequent reduced second-hand smoke, may have contributed to decrease the chronic cell damage and inflammation caused by smoking on GD clinical manifestations; even if the overall prevalence of smokers did not change between centuries, those attempts to limit tobacco use may have contributed to a milder phenotype.

Hyperthyroidism is the main manifestation of GD; in our analysis, hyperthyroidism severity at diagnosis was lower in the post-2000 group, either considering FT4 or FT3 as continuous value or adjusted for upper limit of normal. This result might have different explanations: on one hand, in recent decades, there might be an increased awareness for overall and thyroid health, both in the general population and among general practitioners, leading to an earlier diagnosis, thereby preventing biochemical worsening of uncontrolled hyperthyroidism; on the other hand, mild or subclinical hyperthyroidism at diagnosis might merely express a milder phenotype, remaining stable and not necessarily progressing to overt and more severe forms [22].

Goiter prevalence significantly diminished in the post-2000 cohorts, with meta-regression nicely depicting a progressive volume reduction trend from late 70s to present time, with a plateau in the latest years, yielding an estimated mean thyroid volume at diagnosis of about 35 ml. Goiter evaluation could be biased by the different iodine status in different geographical areas and by different improvement in iodoprophylaxis strategies in recent decades; iodine supplementation is nowadays reported to be optimal in most countries, though 28 countries still have insufficient iodine in their diets [23]. We believe that the worldwide increased awareness of iodine supplementation has been one of the main reasons for the downward trend in goiter prevalence and volume. Considering Italy, a nation with a long history of iodine deficiency, after an effective iodine prophylaxis by iodized salt, urinary iodine excretion has recently reached the cutoff value for iodine sufficiency [24]. Moving from general population to GD cohorts, according to Italian reports, in two studies performed between 1980 and 1990s [25, 26], the prevalence of large goiter (volume > 40 ml) was reported in 52% and 67% of patients, respectively; conversely, in a more recent report, 75% of patients presented no or small goiter at diagnosis [10]. It is conceivable that iodoprophylaxis, especially in iodine-deficient areas, had, at least partially, a role in the diminished goiter prevalence and volume at GD diagnosis observed in this meta-analysis.

GO is the most common extrathyroidal GD manifestation; the estimated GO prevalence in the post-2000 cohort was 25%, significantly lower than 34% of the pre-2000 cohort, meaning that nowadays only one newly diagnosed GD patient out of four have signs and symptoms of GO. This finding is consistent with recent studies, that have also identified a lower severity and activity in most of newly diagnosed GD patients [10, 27]. Unfortunately, we had sparse and heterogeneous data on GO severity; therefore, we could not compare those features in our analysis.

TRAb are the principal etiopathogenic factor of GD, and their evaluation is of fundamental importance both at diagnosis and in the follow-up of the disease [28]; indeed, TRAb persistence or de novo elevation after antithyroid drug therapy discontinuation is a risk factor for relapse of the disease [29]. Many types of immunoassays, both competitive-binding or functional cell-based assays, have been used to measure TRAb throughout the years; therefore, to minimize this bias, on one hand, we assessed TRAb positivity prevalence, according to the given method, as well as serum levels adjusted for upper limit of normal for the specific measurement method employed in the article. Virtually, all patients with GD should test positive for TRAb, but this has not always been the case, mostly due to the low sensitivity of older assays. TRAb prevalence in the post-2000 group was significantly higher than in the pre-2000 group. Furthermore, by meta-regression, we observed a trend toward an increase in TRAb prevalence and levels from the 80s and the mid-90s, and a subsequent plateau and a prevalence of approximately 90% in the latest decades, TRAb levels being on average six times higher than the upper limit of laboratory-specific cutoff values. We believe that this increase is mainly due to the increased sensitivity of immunoassays and bioassays developed and implemented in latest years [30].

Other thyroid autoantibodies are frequently found in GD, but their role is not completely understood. A recent study demonstrated that the presence of TPOAb or thyroglobulin antibodies did not change presenting clinical and biochemical features of GD, but might play a role in transition from hypothyroidism to hyperthyroidism at disease onset, possibly due to the concomitant presence of blocking (or neutral) and stimulating TRAb pool [31]. In our analysis, TPOAb had no relevant role in the phenotype change throughout the decades, since pooled estimated prevalence of TPOAb was around 70%, not different between the pre- and post-2000 groups.

Apart from iodoprophylaxis and worldwide decrease in smoking habits, an intriguing role tempering GD phenotype could have been played by exogenous estrogens, especially considering the high female predisposition for GD; indeed, oral contraceptive pill use has risen in the latest decades [32], and estrogens are considered to be protective against GD [33]. Another interesting, still unproven, possibility is the increased awareness of the importance of micronutrient supplementation, such as vitamin D, which has been reported to be deficient in female patients with GD [34], and selenium, whose supplementation has proven to be beneficial in case of mild GO [35].

Finally, we believe that this milder phenotype has contributed to the change in the therapeutical approach to GD. For instance in the USA, the preferred first-line treatment for GD historically was 131I-radiotherapy; however, the proportion of patients who undergo first-line 131I-radiotherapy is decreasing in USA, with an increased use of thionamides prescriptions [36]. In the end, medical therapy is the first-line therapy for GD worldwide [1]. This circumstance could be due to many reasons, but milder GD phenotype at diagnosis could be seen as a favorable factor to utilize a conservative approach with antithyroid drug as first-line treatment.

Despite our efforts, this meta-analysis and meta-regression has some limitations: (1) though we considered only data from newly diagnosed, untreated patients, there could be a possible selection bias for studies focused on a specific therapy (medical treatment, 131I-radiotherapy or surgery); to limit this issue, in the meta-regression, we adjusted the multivariate analysis for the presence of such therapeutical indications; and (2) as to laboratory findings, assays were heterogeneous; to minimize this bias and standardize our findings we employed different approaches: (1) when data were available only as median and interquartile range, mean and standard deviation were estimated [13]; (2) apart from the raw biochemical level, we considered also the proportion between the mean value compared to the upper limit of normal for the specific measurement method employed in the article; these expedients allowed us to harmonize results, but a certain degree of approximation must be taken into account; and (3) GO data were not reported in all studies, and classification was very heterogeneous; therefore, we decided not to compare and analyze severity of GO, but only its crude prevalence assessed by physical examination.

## Supplementary Information

Below is the link to the electronic supplementary material.

Supplementary file1 (PDF 74 KB)
