# Supplementary material for: Change in newly diagnosed Graves’ disease phenotype between the twentieth and the twenty-first centuries: meta-analysis and meta-regression
Source: J Endocrinol Invest. 2020 Dec 21;44(8):1707–18. doi: 10.1007/s40618-020-01479-z (PMC8285314; doi:10.1007/s40618-020-01479-z)
Supplement: Supplementary file 1 — Supplementary file1 (PDF 74 KB) [file 40618_2020_1479_MOESM1_ESM.pdf]

First Author	Year_Pub	Country	Therapeutical Indication (if any)	Ethnicity	N	Female (%)	Age Mean	Age_SD	Smoke(%)	PMID
Scott	1980	UK	medical therapy	Caucasian	18	89	47	14	N.A.	6894979
Teng	1980	Hong-Kong	radioiodine	Asian	44	66	N.A.	N.A.	N.A.	6103007
Tamai	1980	Japan	medical therapy	Asian	45	87	20	2	N.A.	6892672
Dahlberg	1981	Sweden	medical therapy	Caucasian	54	85	37	12	N.A.	6895351
Sharp	1982	USA-California	medical therapy	Mixed	18	N.A.	43	4	N.A.	6894929
Arteaga	1983	Chile	medical therapy	Hispanic	24	67	34	N.A.	N.A.	6688973
Hegedus	1983	Denmark		Caucasian	90	87	47	11	N.A.	6688972
Romaldini	1983	Brazil-Sao Paulo	medical therapy	Hispanic	113	84	40	12	N.A.	6192139
Bliddal	1983	Denmark		Caucasian	43	84	46	16	N.A.	6139399
Stenszky	1983	Hungary		Caucasian	196	87	38	8	N.A.	6603289
Kawamura	1983	Japan	medical therapy	Asian	61	69	38	12	N.A.	6185525
Ferrari	1983	Italy-Milan		Caucasian	12	N.A.	N.A.	N.A.	N.A.	6687730
Hegedus	1984	Denmark	medical therapy	Caucasian	17	88	42	10	N.A.	6151328
Sridama	1984	USA	medical therapy, radioiodine, surgery	Mixed	432	80	36	11	N.A.	6205272
De Bruin	1984	The Netherlands	medical therapy	Caucasian	32	72	35	14	N.A.	6325045
Hormann	1985	Germany		Caucasian	50	N.A.	N.A.	N.A.	N.A.	2868149
Shen	1985	Taiwan	medical therapy	Asian	5	80	N.A.	N.A.	N.A.	3928675
Schicha	1985	Germany		Caucasian	235	N.A.	N.A.	N.A.	N.A.	3841136
Peden	1985	Scotland	surgery	Caucasian	34	91	39	3	N.A.	3840600
Wilson	1985	Scotland	medical therapy	Caucasian	49	90	37	12	N.A.	2863338
Azizi	1985	USA; Iran	medical therapy	Mixed	51	69	36	10	N.A.	3839244
Roti	1985	Italy; USA	medical therapy	Mixed	16	87	37	5	N.A.	3838710
Eshoj	1985	Denmark	medical therapy	Caucasian	33	82	49	15	N.A.	2859737
Robuschi	1986	Italy-Parma	medical therapy	Caucasian	20	85	39	5	N.A.	3537101
Shiroozu	1986	Japan	medical therapy	Asian	74	69	37	13	N.A.	3011835
Takamtsu	1986	Japan	medical therapy	Asian	47	83	34	11	N.A.	3754263
Weetman	1986	Wales	medical therapy	Caucasian	65	80	38	12	N.A.	2875484
Laurberg	1986	Denmark	medical therapy	Caucasian	99	84	48	12	N.A.	3486110
Wang	1987	Taiwan	medical therapy	Asian	40	N.A.	N.A.	N.A.	N.A.	3654913
Bliddal	1987	Europe		Caucasian	60	85	45	14	N.A.	2888551
Eisenstein	1988	Israel	medical therapy	Arab	29	69	34	10	N.A.	2972741
Mashio	1988	Japan	medical therapy	Asian	54	81	38	14	N.A.	2901181
Murakami	1988	Japan	medical therapy	Asian	18	67	N.A.	N.A.	N.A.	3397034
Nordyke	1988	USA-Hawaii		Mixed	880	N.A.	37	13	N.A.	3341864
Roti	1988	Italy-Parma	medical therapy	Caucasian	22	95	41	4	N.A.	3168311
Klein	1988	USA	medical therapy	Mixed	10	70	35	11	N.A.	3124776
Young	1988	UK		Caucasian	72	87	N.A.	N.A.	N.A.	2902655
Trzepacz	1989	USA		Mixed	25	84	36	12	N.A.	2816972
Aizawa	1989	Japan		Asian	371	80	N.A.	N.A.	N.A.	2794317
Benker	1989	Europe		Caucasian	522	N.A.	N.A.	N.A.	N.A.	2570653
Nagayama	1990	Japan	medical therapy	Asian	50	76	38	15	N.A.	1714371
Nishitani	1990	Japan		Asian	61	100	36	13	N.A.	2258137
Winsa	1990	Sweden	medical therapy, radioiodine	Caucasian	109	86	37	11	N.A.	2375236
Allannic	1990	France	medical therapy	Caucasian	94	85	41	14	N.A.	1689737
Philippou	1991	Greece	medical therapy	Caucasian	17	65	41	3	N.A.	1915648
Hashizume	1991	Japan	medical therapy	Asian	109	78	34	7	N.A.	1900575
Barth	1991	Switzerland	radioiodine	Caucasian	89	95	57	9	N.A.	2071823
Roiter	1991	Italy-Treviso		Caucasian	12	92	N.A.	N.A.	N.A.	1997537
Weryha	1991	France		Caucasian	10	80	40	11	N.A.	1916654
Balasz	1991	Hungary		Caucasian	20	95	44	8	N.A.	1760523
Werner	1991	Brazil-Sao Paulo	medical therapy	Hispanic	49	92	43	13	N.A.	1688155
Tallstedt	1992	Sweden		Caucasian	168	83	40	5	47	1489388
Cho	1992	South Korea	medical therapy	Asian	163	74	40	14	N.A.	1358483
Vitti	1992	Italy-Pisa	medical therapy	Caucasian	105	73	35	13	N.A.	1543017
Nakamura	1993	Japan		Asian	170	85	39	13	N.A.	7920887
Roti	1993	Italy-Parma	medical therapy	Caucasian	18	94	39	4	N.A.	8487661
Rieu	1994	France		Caucasian	53	96	44	15	N.A.	7828357
Burch	1994	USA-Washington DC	radioiodine	Mixed	4	75	29	1	N.A.	7521992
Edmonds	1994	UK	medical therapy	Mixed	95	77	43	12	47	8075780
Takasu	1995	Japan	medical therapy	Asian	182	72	37	17	N.A.	7560811
Rittmaster	1996	Canada	medical therapy	Mixed	70	79	37	15	N.A.	8784084
Torring	1996	Sweden	medical therapy, radioiodine, surgery	Caucasian	179	83	40	5	N.A.	8768863
Kallner	1996	Sweden	medical therapy	Caucasian	94	81	39	10	N.A.	8656146
Prakash	1996	India	medical therapy	Indian	45	84	38	12	N.A.	8925844
Takasu	1997	Japan		Asian	277	74	36	17	N.A.	9364248
Lucas	1997	Spain	medical therapy	Caucasian	60	82	36	11	N.A.	9253309
Abe	1998	Japan	medical therapy, radioiodine, surgery	Asian	287	78	35	11	36	9606287
Mostbeck	1998	Austria		Caucasian	6863	85	47	16	N.A.	9553166
Rittmaster	1998	Canada	medical therapy	Mixed	149	85	38	14	54	9506733
Catargi	1999	France	radioiodine	Caucasian	95	N.A.	52	12	N.A.	10427153
Bringmann	1999	UK	medical therapy	Caucasian	212	80	49	11	N.A.	10342357
Maugendre	1999	France	medical therapy	Caucasian	134	14	42	10	N.A.	10341866
Bogazzi	1999	Italy-Pisa	radioiodine	Caucasian	100	81	48	14	62	10022407
Raber	2000	Austria	medical therapy	Caucasian	114	86	41	12	N.A.	10664518
Glinoer	2001	Belgium	medical therapy	Caucasian	82	85	36	9	14	11331213
Allahabadia	2001	UK	radioiodine	Caucasian	321	79	42	17	N.A.	11502786
Chih-Tsueng He	2003	Taiwan	medical therapy	Asian	30	70	32	7	N.A.	15163329
Ali	2004	Tunisia		Arab	300	71	38	11	N.A.	15026716

Andrade	2004	Brazil	radioiodine	Hispanic	32	88	35	8	47	15476447
Erem	2004	Turkey	radioiodine	Arab	13	85	50	10	N.A.	15545707
Kubota	2005	Japan		Asian	131	74	42	12	N.A.	16410666
Yamada	2006	Japan	medical therapy	Asian	232	89	N.A.	N.A.	N.A.	16983180
Cappelli	2007	Italy-Brescia		Caucasian	216	86	39	13	N.A.	17675761
Alfadda	2007	Saudi Arabia		Arab	194	72	32	13	N.A.	17268701
Corona	2007	Italy-Firenze		Caucasian	8	N.A.	46	21	N.A.	18379127
Cappelli	2008	Italy-Brescia		Caucasian	426	87	50	13	N.A.	17459638
Mazza	2008	Italy-Torino	medical therapy	Caucasian	249	82	52	15	18	19092290
Mao	2009	China		Asian	191	86	35	11	N.A.	19850692
Vos	2009	The Netherlands		Caucasian	251	75	42	13	N.A.	19423562
Niyerenda	2009	USA-Tennessee		Caucasian	59	97	40	15	42	19141763
Cinemre	2009	Turkey	medical therapy	Caucasian	26	85	47	16	23	19480735
Takata	2010	Japan	medical therapy	Asian	134	72	39	14	N.A.	199122243
Gupta	2011	USA-Ohio		Caucasian	20	90	38	11	50	11716044
Vieira	2011	Brazil		Hispanic	103	80	42	12	N.A.	22231972
Anagnostis	2012	Greece		Caucasian	211	79	47	9	65	23397523
Li	2012	China	medical therapy	Asian	60	82	32	5	N.A.	22186223
Tanda	2013	Italy-Varese, Pavia		Caucasian	346	77	46	14	35	23408569
Dauksiene	2013	Lituania		Caucasian	194	80	42	12	N.A.	24709784
Mohlin	2013	Sweden	medical therapy	Caucasian	291	83	46	N.A.	32	24366943
Lantz	2014	Sweden		Caucasian	399	N.A.	N.A.	N.A.	N.A.	24554511
Chen	2014	China		Asian	288	71	34	12	8	24203649
Vaidya	2014	UK	medical therapy	Caucasian	450	82	49	16	15	24801484
Goichot	2015	France		Caucasian	802	81	43	14	23	25959282
Sato	2015	Japan	medical therapy	Asian	310	80	40	15	N.A.	25178068
Diagne	2016	Senegal		African	108	88	38	14	N.A.	28154699
Magri	2016	Italy-Pavia		Caucasian	294	78	46	15	35	27540886
Bartalena	2016	Italy-Varese, Pavia		Caucasian	283	75	48	14	31	27465670
Martin	2016	Romania		Caucasian	80	81	44	16	36	18046061
Lanas	2017	Chile		Hispanic	212	76	44	18	N.A.	28748990
Sundaresh	2017	USA-Minnesota		Caucasian	720	77	49	15	17	28049375
